# Dietary, Nutrient Patterns and Blood Essential Elements in Chinese Children with ADHD

**DOI:** 10.3390/nu8060352

**Published:** 2016-06-08

**Authors:** Fankun Zhou, Fengyun Wu, Shipu Zou, Ying Chen, Chang Feng, Guangqin Fan

**Affiliations:** 1 State Key Laboratory of Food Science and Technology, Nanchang University, Nanchang 330047, China; zhoufankun66@163.com; 2 Department of Occupational Health and Toxicology, School of Public Health, Nanchang University, Nanchang 330006, China; waterncu@126.com (F.W.); goldncu@163.com (Y.C.); gloriqina@163.com (C.F.); 3 Department of Child Healthcare, Children’s Hospital of Jiangxi Province, Nanchang 330006, China; zoushipujxch@126.com

**Keywords:** ADHD, dietary pattern, nutrient pattern, blood essential elements, Chinese children

## Abstract

Dietary or nutrient patterns represent the combined effects of foods or nutrients, and elucidate efficaciously the impact of diet on diseases. Because the pharmacotherapy on attention deficit hyperactivity disorder (ADHD) was reported be associated with certain side effects, and the etiology of ADHD is multifactorial, this study investigated the association of dietary and nutrient patterns with the risk of ADHD. We conducted a case-control study with 592 Chinese children including ADHD (*n* = 296) and non-ADHD (*n* = 296) aged 6–14 years old, matched by age and sex. Dietary and nutrient patterns were identified using factor analysis and a food frequency questionnaire. Blood essential elements levels were measured using atomic absorption spectrometry. A fish-white meat dietary pattern rich in shellfish, deep water fish, white meat, freshwater fish, organ meat and fungi and algae was inversely associated with ADHD (*p* = 0.006). Further analysis found that a mineral-protein nutrient pattern rich in zinc, protein, phosphorus, selenium, calcium and riboflavin was inversely associated with ADHD (*p* = 0.014). Additionally, the blood zinc was also negatively related to ADHD (*p* = 0.003). In conclusion, the fish-white meat dietary pattern and mineral-protein nutrient pattern may have beneficial effects on ADHD in Chinese children, and blood zinc may be helpful in distinguishing ADHD in Chinese children.

## 1. Introduction

Attention deficit hyperactivity disorder (ADHD), one of the most common childhood psychiatric disorders, is a worldwide public problem that is characterized by developmentally inappropriate levels of inattention, impulsivity, and hyperactivity [1,2]. This disorder in children can severely affect their school performance, family relationships, and social interactions [3]. Currently, the treatment of ADHD is mainly dependent on the pharmacotherapy that involves stimulant medications, such as methylphenidate, amphetamine, and atomoxetine, which has shown a high effectiveness in ADHD treatment [4,5,6,7], but it has been reported by several studies to be associated with certain side effects [6,7,8]. The etiology of ADHD is multifactorial, and diet is an influencing factor of ADHD that is amenable to modification [9].

Based on current studies, diets that reduce the symptoms associated with ADHD include restricting or eliminating the detrimental dietary factors that are associated with the risk of ADHD and supplementing the beneficial dietary factors that protect against ADHD [10,11,12,13,14,15,16]. For the detrimental dietary factors, the proposed dietary treatments include restricting sugar and ensuring additive/preservative-free foods (Feingold Diet) and an oligoantigenic diet (elimination) [11,12,13]. However, restricting or eliminating the detrimental dietary factors is complicated, time-consuming, disruptive to the household, and often impractical, except for selected patients. For the beneficial dietary factors, the proposed dietary treatments include polyunsaturated fatty acids (PUFAs) or omega-3 supplements, minerals such as iron and zinc supplements, as well as a megavitamin supplement diet. Supplemental diet therapy is simple, relatively inexpensive, and more acceptable to the patients and parents [10,14,15,16].

Foods and nutrients are consumed in various characteristic combinations that synergistically influence the risks related to many diseases. Dietary or nutrient patterns represent a broader series of food and nutrient consumption that is more predictive of disease risk than individual foods or nutrients [17]. In recent years, researchers have transferred their focus from individual food or nutrition factors to dietary patterns. Some studies suggested that a healthy diet pattern and lifestyle to prevent or control ADHD might have greater long-term success [10,18,19,20]. Isolated studies have reported on the relationships between dietary patterns and ADHD [18,19,20]. Howard *et al.* and Azadbakht *et al.* have examined unhealthy dietary patterns associated with ADHD in their cross-sectional studies [18,19]. In the adolescents of Western Australia, Howard *et al.* reported that a Western-style dietary pattern consisting of a high intake of fat, refined sugars, and sodium and a low intake of fiber, folate, and omega-3 fatty acids was associated with increased odds of an ADHD diagnosis, whereas a healthy dietary pattern, with a high intake of fiber, folate, and omega-3 fatty acids, was not correlated with the diagnosis of ADHD [18]. In Iranian school-aged children, dietary patterns characterized by a high intake of sweets and fast food were associated with a greater risk of ADHD; however, no significant association was observed for the healthy or Western dietary patterns [19]. The above cross-sectional investigations indicated the association between dietary patterns and ADHD. A recent further analytical study conducted in Korean school-aged children by Woo *et al.* studied the likely causal relationship between dietary patterns and ADHD using factor analysis and 24 h diet recall (24HR) interview based on a case-control study [20]. The results showed that a traditional, healthy dietary pattern that was characterized by a high intake of kimchi, grains, and bonefish and a low intake of fast foods and beverage, was inversely associated with ADHD [20]. However, the traditional foods or eating habits were different in different regions, and a food frequency questionnaire is more suitable for researching the relationship between diet intake and chronic diseases, including ADHD [21]. Thus, the association between the dietary patterns and ADHD, especially nutrient patterns that have been not researched in the previous studies, should be further explored to provide a scientific basis for preventing or controlling ADHD. In the current research, a matched case-control study was conducted to investigate the dietary patterns and nutrient patterns using factor analysis and a food frequency questionnaire among Chinese school-aged children with ADHD. In addition, an association between the blood essential elements (iron, zinc, calcium, copper and magnesium) and ADHD was evaluated in Chinese children.

## 2. Methods and Materials

### 2.1. Study Population and Recruitment

The matched case-control study participants were consecutively recruited from children attending pediatric clinics for health care and ADHD assessment from the Jiangxi Provincial Children’s Hospital in Jiangxi Province, China. This hospital, which is the largest hospital that specifically treats children in Jiangxi province, serves children from all of the administrative districts within the province.

The subjects with ADHD 10th revision of the International Statistical Classification of Diseases and Related Health Problems (ICD-10 codes F90, 208–210) were children of Chinese Han nationality who were aged 6–14 years at the time of the investigation and had a lifetime medical history that fulfilled the Diagnostic and Statistical Manual of Mental Disorders, 4th ed., revised Diagnostic and Statistical Manual of Mental Disorders, 4th ed. (DSM-IV-R) criteria for ADHD [1]. The individuals were excluded for any evidence of identifiable prenatal insults, autism, Asperger syndrome, epilepsy or a primary diagnosis of schizophrenia, affective disorder, pervasive developmental disorder (ICD-10 codes F84.0–F84.9, 308.0), and mental retardation (ICD-10 codes F70–F79, 312–315).

The non-ADHD control children were randomly selected from those receiving routine health care at the same clinics where the ADHD cases were diagnosed. The controls were given the same full diagnostic assessment as the ADHD cases and screened only for the absence of ADHD without exclusion of any other diagnosis except for the same exclusion criteria applied to ADHD cases. By the pair-matched design, each subject with ADHD and control set had the same sex and the same age (difference between birthdays was within six months).

The face-to-face interviews were performed in the outpatient clinic by graduate students in epidemiology, and the ADHD diagnosis was made by experienced board-certified child psychiatrists. The design and conduct of the survey was performed according to the guidelines laid down in the Declaration of Helsinki, and all of the procedures involving human subjects/patients were approved by the Institutional Review Board of the First Affiliated Hospital of Nanchang University. Informed consent was obtained from the parents of all of the children before participation.

### 2.2. Data Collection

The parents of the participating subjects with ADHD and controls were interviewed in person by the study personnel. All of the personnel were current graduate students in epidemiology. Each interviewer was trained in epidemiologic interviewing, dietary assessment, and anthropometry. All of the interviews with the parents of subjects with ADHD and controls were conducted by two individuals under similar conditions. The data were collected using a standardized and structured questionnaire by individuals who were blinded to the subject or control status of the participants. The data collected included demographic information and other relevant factors related to their children, including the family structure, home environment, socioeconomic status, maternal and paternal education, maternal age at childbirth, smoking behavior of the parents, and medical, obstetric, neuro-developmental, and educational histories of the children, as well as the dietary habits in the 12 months before diagnosis (ADHD-patient) or interview (control subjects). The body mass index (BMI) was calculated (kg/m^2^) based on weight and height measurements with the subjects wearing light clothes and no shoes, according to standard protocols by the World Health Organization (WHO) [22].

### 2.3. Dietary Assessment and Food Grouping

Dietary intake was assessed by a food frequency questionnaire (FFQ), which consisted of 144 food items that were commonly consumed by the Chinese children. All of the questionnaires used for data collection were designed by a panel of experts, including scientists in the fields of epidemiology and nutrition. For each food item, the parents were invited to indicate their children’s average frequency of consumption over the past year in terms of the specified serving size by checking one of nine frequency categories, which ranged from “almost never” to “≥6 times/day” [23]. Photographs of the food portion sizes were provided to help estimate the amount of food consumption. The selected frequency category for each food item was converted to a value in number of servings per day. Portion sizes of consumed foods were converted to grams (g) by using household measurements. To reduce the complexity of the data, we categorized the 144 items into 28 food groups based on the similarities between the nutrient profiles or processing methods, as shown in Table A1, regarding dietary pattern analyses. The average daily intake of nutrients and total energy were calculated according to the Chinese Standard Food Composition Table [24,25]. The questionnaires were excluded from additional analyses if energy intake was implausible (<700 kcal/day or >6000 kcal/day), corresponding to the percentiles <2.5% or >97.5%, respectively.

The reliability of the FFQ in this study was evaluated in a randomly chosen subgroup of 62 children by comparing nutrient consumption determined by responses on the FFQ on two occasions. The FFQ had a high reliability for nutrients. Mean value for intake of total energy and most nutrients assessed by the FFQ on two occasions were not statistically different. Energy-adjusted correlation coefficients for the principal macronutrients ranged from 0.38 for proteins to 0.65 for zinc. Comparative validity was determined by comparison with intakes estimated from the average of six non-consecutive 24 h dietary recalls. We found that most nutrients were moderately correlated (all correlation coefficients >0.4) between these two methods after controlling for total energy intake. Overall, these data indicated that the FFQ provides reasonably valid measurements of average long-term dietary intake.

### 2.4. Blood Sampling and Analysis

Venous whole blood samples (2 mL/child) were collected from the cubital vein using a metal-free and heparinized vacuum tube. The concentrations of blood iron, zinc, calcium, copper and magnesium were measured using atomic absorption spectrometry (BH5100, Beijing Optoelectronic Technology Co., Ltd., Bo Hui innovation, Beijing, China). The detection limits for iron, zinc, calcium, copper and magnesium were 6.0 µg/L, 1.0 µg/L, 3.0 µg/L, 1.0 µg/L and 0.1 µg/L, respectively. The values were the means of four analyses of each sample. The quality control measures showed the inter-run coefficients of variation of 3.0%–5.0% for these elements.

### 2.5. Covariates

Sex and age are associated with the prevalence of ADHD, which was demonstrated in several epidemiologic studies [26,27]. To address these important confounding factors, we employed a pair-match design on age and sex; thus, the stratified control subjects were at risk at the same sex and age.

Additionally, we considered multiple covariates and potential confounders for the association of dietary patterns, nutrient patterns, blood elements and ADHD in our study. They were based on the established predictors of child behavioral problems [26,28,29]. The following variables were used: perinatal distress (low birth weight and admission to a neonatal intensive care unit as markers), household composition (normal: child lives with biological parents; single: child lives with only one parent; or recombined: child lives in remarried family), family history of ADHD (ADHD in parents and siblings, diagnosed by psychiatrists, obtained from clinical reports), maternal smoking during pregnancy (at least one cigarette per day during the last trimester), maternal drinking during pregnancy (at least two glasses per week during the entire pregnancy), paternal smoking at the time of childbirth, labor complications, maternal abortion history, maternal age at childbirth, parents’ education, household income, breast feeding, emotional abuse, and family conflicts. These variables were obtained from the clinical records or questionnaires completed by direct interviews with the parents.

### 2.6. Statistical Analysis

We used paired *t*-tests (continuous variables) or paired χ^2^ tests (categorical variables) to compare the demographic characteristics and other ADHD risk factors between the subjects with ADHD and controls. Factor analysis was adopted to derive the major dietary patterns on the basis of the 28 food groups [30]. To identify the number of factors to be retained, we used the criterion of eigenvalues >1.0, scree test, and interpretability [31]. Finally, four major dietary patterns were identified. Those factors were rotated by orthogonal transformation (VARIMAX procedure) to achieve a simpler structure with greater interpretability. Factor loadings represent correlation coefficients between individual food groups and dietary patterns, and those food groups having high absolute loadings (≥0.3) were considered to be the primary contributors [32]. The proportion of variance explained by each factor was calculated by dividing the sum of the squares of the respective factor loadings by the number of variables. The factor scores for each pattern and for each individual were determined by summing the intakes of each food group weighted by the factor loading [31]. The scores were used for comparison with dietary intake and were divided into tertiles (T1–T3) according to the distribution of factor scores among the controls to estimate their associations with ADHD. Energy-adjusted means for dietary variables across quintiles of dietary pattern scores were calculated. Energy adjustment by using the residual method was performed after entering the food groups in the factor analysis and after analyzing the dietary pattern [33]. The same methods were used to analyze the nutrient patterns, and four major nutrient patterns were identified.

The conditional logistic regressions were used to evaluate the association between the dietary and nutrient patterns and ADHD. For each pattern, T1 was considered to be the reference. The univariate and multivariate analyses were applied to calculate the odds ratios (ORs) and their corresponding 95% confidence intervals (CIs). In the univariate analyses, the crude ORs were calculated without any adjustment in the first model. In the multivariate analyses, we made adjustments for the other potential confounding factors (including BMI, energy intake, family history of ADHD, household composition, maternal smoking during pregnancy, maternal drinking during pregnancy, paternal smoking before childbirth, paternal smoking after childbirth, maternal abortion history, maternal age at childbirth, parents’ education, household income, labor or delivery complications, birth weight, twin, full-term pregnancy, breast feeding, emotional abuse, and family conflicts). All of the covariates were introduced using the forward stepwise method. To test for linear trends, the dietary pattern groups for each factor were treated as continuous variables. We also performed a conditional logistic regression analysis with a binary outcome of ADHD in relation to the blood element levels. In addition, the ORs and 95% CIs of ADHD for each blood element were then calculated as the risk for a change in the blood element level by one standard deviation (SD). Meanwhile, adjustments for other potential confounding factors were made.

SPSS software (version 17.0, Chicago, IL, USA) was used for all of the statistical analyses, and *p* < 0.05 was considered to be statistically significant.

## 3. Results

Of the 340 children with ADHD diagnosed in pediatric clinics, 11 (3.2%) did not meet the selection criteria for this study. Of the 329 eligible subjects, 33 (10.0%) were excluded because of refusal to participate or unreasonable energy intakes (reasonable range, 700–6000 kcal/day). Finally, a total of 296 ADHD subjects were retained in this analysis, including 248 boys and 48 girls. Additionally, 296 eligible age- (±6 months) and gender-matched controls were recruited from the same pediatric clinics.

The demographics and other characteristics are shown in Table 1. Compared with the controls, the subjects with ADHD were more likely to suffer from emotional abuse, have low maternal education and maternal abortion history along with a higher likelihood of a family history of ADHD, and were unlikely to have a household with two biological parents or full-term pregnancy. The subjects with ADHD and controls did not differ in BMI, breast feeding, birth weight, labor or delivery complications, twin, household income, maternal age at childbirth, paternal education, family conflicts, maternal drinking during pregnancy, maternal smoking during pregnancy, paternal smoking before childbirth, and paternal smoking after childbirth and energy intake.

For dietary pattern analysis, four primary factors were retained from the factor analysis. The factor loadings associated with each pattern are shown in Table 2. The high positive loadings indicate strong associations between given food groups and patterns, whereas the negative loadings indicate negative associations with the patterns. Each pattern was labeled according to the food groups with high absolute loadings (≥0.3). Factor 1, with high loadings for leafy vegetables, fruiting vegetables, tuber vegetables, fruits, and root vegetables was labeled as the “vegetable-fruit” dietary pattern. Factor 2, with high loadings for shellfish, deep water fish, white meat, freshwater fish, organ meat, and fungi and algae was labeled as the “fish-white meat” dietary pattern. Factor 3, with high loadings for process grains, soya beans, grains, and other beans, but low loadings for drinks was labeled as the “grain-bean” dietary pattern. Factor 4, with high loadings for fast food, ice cream, sweets, snacks, and red meat, but low loadings for dairy products was labeled as the “fast food-sweet” dietary pattern. Overall, the four dietary patterns accounted for 32.50% of the variance in food intake. The univariate conditional logistic regression analyses showed a dose-dependent inverse association between the risk of ADHD and the fish-white meat dietary pattern (*p* trend = 0.003) and grain-bean dietary pattern (*p* trend = 0.018) (Table 3). With adjustments made in model 2 for other potential confounding factors, significant negative relationships were observed between the risk of ADHD and the fish-white meat dietary pattern (T3 *vs.* T1: OR, 0.44; 95% CI, 0.27–0.73; *p* trend = 0.006). No association with ADHD was observed for any other dietary pattern (Table 3).

Moreover, the nutrient patterns and factor loading scores are presented in Table 4. Factor 1, with high loadings for isoleucine, valine, phenylalanine, leucine, histidine, threonine, lysine, tryptophan, carbohydrate, methionine, was labeled as the “amino acid-carbohydrate” nutrient pattern. Factor 2, with high loadings for folic acid, vitamin B_6_, dietary fiber, magnesium, iron, potassium, vitamin A, vitamin E, thiamine, copper, niacin, choline and vitamin C, was labeled as the “vitamin-dietary fiber” nutrient pattern. Factor 3, with high loadings for zinc, protein, phosphorus, selenium, calcium and riboflavin, was labeled as the “mineral-protein” nutrient pattern. Factor 4, with high loadings for total fat, polyunsaturated fatty acids, saturated fatty acids, monounsaturated fatty acids and cholesterol, but low loadings for methionine, manganese, and copper, was labeled as the “fatty acid-cholesterol” nutrient pattern. Overall, the four nutrient patterns amounted to 65.72% of the variance in food intake. We analyzed the ORs (95% CIs) of tertiles of nutrient patterns and ADHD (Table 5). The highest tertile of the mineral-protein nutrient pattern (T3 *vs.* T1: OR, 0.53; 95% CI: 0.32–0.88; *p* trend = 0.014) was negatively associated with the risk of ADHD after adjusting for other potential confounding factors. However, the amino acid-carbohydrate, vitamin-dietary fiber and fatty acid-cholesterol nutrient patterns were not significantly associated with the risk of ADHD.

The associations between blood element (iron, zinc, calcium, copper and magnesium) levels and ADHD were conducted using conditional logistic regression. The concentration of blood zinc was significantly inversely associated with the risk of ADHD, as shown in model 1 of Table 6 (*p* = 0.005; 95% CI for OR was 0.96–0.99). After an adjustment was made in model 2 for other potential confounding factors, inversely significant associations existed between the risk of ADHD and blood zinc in Table 6 (*p* = 0.008; 95% CI for OR was 0.96–0.99). In the fully adjusted model, one SD increase of blood zinc concentration was associated with an OR of 0.73 (95% CI 0.60–0.89) for ADHD. However, no relation was observed between blood iron, calcium, copper, or magnesium concentration and ADHD (*p* > 0.05).

## 4. Discussion

In the present case-control study, we identified four major dietary patterns and four major nutrient patterns for Chinese children. A fish-white meat dietary pattern rich in shellfish, deep water fish, white meat, freshwater fish, fungi and algae and organ meat was inversely associated with ADHD. Further analysis found that a mineral-protein nutrient pattern rich in zinc, protein, phosphorus, selenium, calcium and riboflavin was inversely associated with ADHD. Additionally, the blood zinc was also negatively related to ADHD. To the best of our knowledge, this study, with 296 pairs of age- and gender-matched Chinese children with ADHD and controls, is the first to specifically identify the potential influence of nutrient patterns on the risk of ADHD based on the association between dietary patterns and ADHD.

The diet pattern (including dietary pattern and nutrient pattern) is an aggregate variable that is derived from special statistical methods, which summarizes the food and nutrient intake to create a meaningful overall representation of a large and complex set of interrelated diet factors. Recently, diet pattern analysis has emerged in the field of nutritional epidemiology as an alternative and complementary approach for examining the relationship between diet and the risk of chronic diseases. Rather than considering individual foods or nutrients, diet patterns conceptually represent a broader picture of food and nutrient consumption and may thus be more predictive of disease risk than individual foods or nutrients [34]. Moreover, a healthy diet pattern may be an effective and practical method to prevent or control ADHD.

To date, three previous studies have examined the associations between dietary patterns and ADHD [18,19,20]. These studies and our current research all suggested that several special dietary patterns were associated with ADHD. It is interesting that the healthy dietary patterns, which were significant, protectively related to ADHD in the case-control studies of Woo *et al.* and ours, included vegetable, fresh fruit, fish, white meat, and whole grains [20], whereas the unhealthy dietary patterns that were significant, adversely related to ADHD in the cross-sectional studies of Howard *et al.* and Azadbakht *et al.*, included fast food, ice cream, sweets, snacks, and red meat [18,19].

To further explore the relationship of dietary patterns and ADHD, a nutrient pattern analysis was conducted in our study. In addition, a mineral-protein nutrient pattern typified by the consumption of zinc, protein, phosphorus, selenium, riboflavin and calcium was associated with a 53% (95% CI, 29%–68%) decrease in the risk of ADHD (T3 *vs.* T1). This finding interestingly supported the relationship of fish-white meat pattern and ADHD, because the fish and white meat were abundant with zinc, selenium, calcium, phosphorus and purified protein [24,25]. The results of Howard *et al.* and Azadbakht *et al.* also supported our finding on the opposite side [18,19]. Their unhealthy dietary patterns such as fast food consumption were found to be associated with having a lower intake of mineral nutrients, including zinc, calcium and phosphorus [35,36]. However, it was not found in our study that the PUFAs abundantly contained in fish were associated with ADHD [14,37], which was reported by some studies. In our nutrient factor analysis, saturated fatty acids and polyunsaturated fatty acids were always loaded together in the fatty acid-cholesterol nutrient pattern. We regarded that the association between dietary patterns and ADHD might be mainly attributed to the minerals in the mineral-protein nutrient pattern.

To further verify and elucidate the relation between minerals and ADHD in our study, the essential elements zinc, iron, calcium, copper and magnesium that were closely related to children's growth and development were detected in children’s blood [38]. The results showed that blood zinc was inversely associated with ADHD and a lower zinc level was discovered in the ADHD children. These findings corresponded with the above nutrient pattern results. The same results were also discovered in the studies conducted by Akhondzadeh *et al.* [39] and Bilici *et al.* [15], which also found that children with ADHD are associated with zinc deficiency, and zinc supplementation can reduce ADHD symptoms in children with low zinc levels. It is known that zinc is necessary for many metal-enzyme complexes, lots of them located in the nervous system [40], and that it contributes to construction and function of the brain [41]. Zinc is also essential for conversion of dietary pyridoxine to its active form, pyridoxal phosphate, and pyridoxine is necessary for conversion of tryptophan to serotonin, which had a close relationship with ADHD [15,42]. In addition, zinc is basic for the production and modulation of melatonin, which helps regulate dopamine function, and is supposed to be an important factor in ADHD [43,44]. Therefore, zinc played a major role in the relationships between minerals and ADHD in our study, and the blood zinc was helpful for distinguishing ADHD in children.

Some points need to be considered in the interpretation of our findings. First, many well-known lifestyle factors and other risk factors for ADHD were significantly different between the subjects with ADHD and the controls (e.g., family history of ADHD, emotional abuse, full-term pregnancy, household composition, maternal education, and maternal abortion history). Although we adjusted for many such factors using statistical analysis, residual confounding was still unavoidable because of measurement errors and missing adjustments for several unmeasured factors. Second, the FFQs are known to contain a certain degree of measurement error although the FFQ used in our study has been previously validated. Third, the four major dietary patterns derived from our data explained only 32.5% of total variance, suggesting the existence of other dietary patterns. However, these values should be interpreted with caution because they depend heavily on the total number of variables used in the factor analysis [31]. Finally, the studies with hospital-based controls are prone to selection bias, which might confound the results.

## 5. Conclusions

In conclusion, our findings suggest that the consumption of a fish-white meat dietary pattern and a mineral-protein nutrient pattern may have beneficial effects on ADHD, and may be effective and practical methods to prevent or control ADHD in Chinese children. Additionally, blood zinc might be helpful to distinguish ADHD in Chinese children. Further prospective studies are required to confirm these findings.

## Figures and Tables

**Table 1:** Demographic and distribution of the risk factors of ADHD and controls ^1^.

Characteristic	ADHD (*n* = 296)	Controls (*n* = 296)	*p* ^2^
*Matched Factors*			
Age (years)	8.42 ± 1.72	8.46 ± 1.84	-
Gender			-
Male	248 (83.8)	248 (83.8)	
Female	48 (16.2)	48 (16.2)	
*Child Factors*			
BMI (kg/m^2^)	16.00 ± 3.00	16.41 ± 2.93	0.263
Energy intake (kcal/day)	1778.4 ± 508.1	1895.7 ± 631.9	0.681
Breast feeding	172 (58.1)	182 (61.5)	0.225
Emotional abuse	180 (60.8)	101 (34.1)	**<0.001**
Birth weight (kg)	3.27 ± 0.56	3.24 ± 0.52	0.436
Full-term pregnancy	254 (85.8)	273 (92.2)	**0.017**
Labor or delivery complications	29 (9.8)	21 (7.1)	0.150
Twin	16 (5.4)	14 (4.7)	0.426
Family history of ADHD	40 (13.5)	21 (7.1)	**0.007**
*Family Environmental Factors*			
Household composition			**0.011**
Two parents	210 (70.9)	241(81.4)	
Single parent	20 (6.8)	17 (5.7)	
Recombined	66 (22.3)	38 (12.9)	
Household income (yuan/month/person)			0.922
≤1000	95 (32.1)	93 (30.7)	
1000–2000	99 (33.4)	99 (33.4)	
≥2000	102 (34.5)	106 (35.8)	
Maternal age at childbirth	25.39 ± 3.46	25.55 ± 3.60	0.609
Maternal education (years)			**0.032**
≤9	37 (12.5)	55 (18.6)	
9–12	126 (42.6)	101 (34.1)	
≥12	133 (44.9)	140 (47.3)	
Paternal education (years)			0.229
≤9	22 (7.4)	28 (9.5)	
9–12	116 (39.2)	102 (34.5)	
≥12	158 (53.4)	166 (56.0)	
Maternal abortion history	107 (36.1)	76 (25.7)	**0.004**
Family conflicts	11 (3.7)	10 (3.4)	0.500
Maternal drinking during pregnancy	3 (1.0)	3 (1.0)	0.657
Maternal smoking during pregnancy	2 (0.7)	1 (0.3)	0.500
Paternal smoking before childbirth	151 (51.0)	141 (47.6)	0.230
Paternal smoking after childbirth	372 (40.2)	101 (34.1)	0.074

-: no data; ADHD: attention deficit hyperactivity disorder; BMI: body mass index; ^1^: Categorical variables are expressed as *n* (%); the continuous variables are expressed as the mean ± SD values; ^2^: Statistically significant *p* values are indicated in bold.

**Table 2:** Factor loading matrix for the four identified dietary patterns in the study children ^1^.

Food Groups or Foods ^2^	Dietary Patterns			
	Factor 1 (Vegetable-Fruit)	Factor 2 (Fish-White Meat)	Factor 3 (Grain-Bean)	Factor 4 (Fast Food-Sweet)
Leafy vegetables	**0.71**	0.01	0.11	0.02
Fruiting vegetables	**0.68**	0.03	0.24	0.01
Tuber vegetables	**0.67**	0.07	0.10	−0.01
Fruits	**0.47**	0.21	−0.12	−0.02
Root vegetables	**0.35**	0.00	0.25	−0.05
Shellfish	−0.13	**0.65**	0.10	−0.05
Deep water fish	−0.12	**0.58**	0.11	−0.10
White meat	0.04	**0.54**	−0.05	0.02
Freshwater fish	0.08	**0.51**	0.02	−0.15
Organ meat	0.00	**0.50**	−0.06	0.05
Fungi and algae	0.21	**0.42**	0.10	−0.02
Condiments	0.12	0.24	−0.07	0.13
Animal fats	−0.01	−0.24	−0.11	0.02
Nuts	0.14	0.14	0.05	−0.02
Process grains	−0.14	−0.22	**0.69**	0.21
Soya beans	0.10	0.03	**0.62**	0.01
Grains	−0.14	−0.12	**0.57**	−0.15
Drinks	−0.07	0.06	**−0.53**	0.22
Other beans	0.22	0.10	**0.41**	0.04
Vegetable oils	−0.10	0.07	−0.24	0.11
Tuber crops	0.14	0.09	0.24	−0.04
Fast food	0.14	−0.08	−0.15	**0.73**
Ice cream	0.07	−0.09	−0.16	**0.60**
Sweets	0.17	0.07	−0.16	**0.49**
Snacks	0.16	−0.21	−0.16	**0.47**
Dairy products	0.17	−0.16	−0.01	**0.32**
Red meat	−0.14	0.12	0.08	−0.01
Eggs	0.71	0.01	0.11	0.02
Proportion of explained variance (%)	8.57	8.49	8.23	7.21

^1^: Dietary patterns were determined by factor analysis; ^2^: The factor-loading scores with absolute values of ≥0.30 are bolded.

**Table 3:** Odds ratio (95% CIs) of ADHD for tertiles of dietary patterns ^1^.

	Tertiles of Dietary Energy-Adjusted Intake			*p* for Trend
	T1 (Referent)	T2	T3 (Highest)	
Factor 1 (vegetable-fruit dietary pattern)				
*n* (ADHD/control)	108/90	94/103	94/103	
Model 1	1.00	0.76 (0.51, 1.13)	0.76 (0.51, 1.13)	0.293
Model 2	1.00	0.72 (0.43, 1.19)	0.69 (0.42, 1.13)	0.274
Factor 2 (fish-white meat dietary pattern)				
*n* (ADHD/control)	118/80	90/107	88/109	
Model 1	1.00	0.57 (0.38, 0.85)	0.55 (0.37, 0.82)	0.003
Model 2	1.00	0.64 (0.38, 1.06)	0.44 (0.27, 0.73)	0.006
Factor 3 (grain-bean dietary pattern)				
*n* (ADHD/control)	114/84	92/105	90/107	
Model 1	1.00	0.65 (0.43, 0.96)	0.62 (0.42, 0.92)	0.018
Model 2	1.00	0.61 (0.37, 1.01)	0.60 (0.36, 1.05)	0.087
Factor 4 (fast food-sweet dietary pattern)				
*n* (ADHD/control)	91/106	95/102	110/88	
Model 1	1.00	1.09 (0.73, 1.61)	1.46 (0.98, 2.16)	0.148
Model 2	1.00	0.94 (0.57, 1.57)	1.25 (0.76, 2.06)	0.490

Models 1 and 2: from conditional logistic model. Model 1: without further adjustment; Model 2: covariates adjusted for other potential confounding factors, including BMI, energy intake, family history of ADHD, household composition, maternal smoking during pregnancy, maternal drinking during pregnancy, paternal smoking before childbirth, paternal smoking after childbirth, maternal abortion history, maternal age at childbirth, parents’ education, household income, labor or delivery complications, birth weight, twin, full-term pregnancy, breast feeding, emotional abuse, and family conflicts; ^1^: ORs (95% CI) for all such values.

**Table 4:** Factor loading matrix for the four identified nutrient patterns in the study children ^1^.

Nutrient ^2^	Nutrient Patterns			
	Factor 1 (Amino Acid-Carbohydrate)	Factor 2 (Vitamin-Dietary Fiber)	Factor 3 (Mineral-Protein)	Factor 4 (Fatty Acid-Cholesterol)
Isoleucine	**0.76**	0.15	0.16	0.07
Valine	**0.76**	0.14	0.20	0.05
Phenylalanine	**0.75**	0.18	0.12	0.03
Leucine	**0.74**	0.16	0.18	0.06
Histidine	**0.64**	0.20	0.19	0.08
Threonine	**0.63**	0.13	0.22	0.09
Lysine	**0.52**	0.16	0.19	0.10
Tryptophan	**0.51**	0.21	0.10	−0.20
Carbohydrate	**0.50**	−0.10	−0.22	−0.20
Methionine	**0.34**	0.13	0.24	−0.47
Folic acid	0.06	**0.64**	0.06	0.15
Vitamin B_6_	0.23	**0.62**	−0.08	0.09
Dietary fiber	0.21	**0.58**	0.12	−0.01
Magnesium	0.16	**0.51**	0.23	−0.15
Iron	0.23	**0.43**	0.24	−0.18
Potassium	0.19	**0.42**	0.24	−0.13
Vitamin A	−0.05	**0.40**	0.18	0.07
Vitamin E	0.15	**0.40**	0.02	0.17
Thiamine	0.19	**0.34**	0.20	−0.07
Copper	0.22	**0.34**	0.21	−0.31
Niacin	0.02	**0.33**	0.17	−0.18
Choline	0.21	**0.32**	0.04	0.16
Vitamin C	−0.04	**0.32**	0.06	−0.14
Iodine	0.16	0.20	−0.06	0.17
Sodium	−0.08	0.09	0.09	0.08
Zinc	0.19	0.23	**0.59**	−0.17
Protein	0.21	0.18	**0.54**	−0.16
Phosphorus	0.21	0.16	**0.50**	0.01
Selenium	0.03	0.11	**0.49**	−0.16
Riboflavin	0.06	0.03	**0.47**	0.13
Calcium	0.24	0.07	**0.42**	0.03
Total fat	−0.11	−0.15	0.16	**0.57**
Polyunsaturated fatty acids	0.16	0.08	0.18	**0.53**
Saturated fatty acids	0.03	−0.10	−0.25	**0.43**
Monounsaturated fatty acids	0.18	0.16	0.01	**0.39**
Cholesterol	0.16	−0.16	0.13	**0.34**
Manganese	0.18	0.19	0.18	**−0.32**
Proportion of explained variance (%)	24.99	16.93	12.90	10.38

^1^: Dietary patterns were determined by factor analysis; ^2^: The factor-loading scores with absolute values of ≥0.30 are bolded.

**Table 5:** Odds ratio (95% CIs) of ADHD for tertiles of nutrient patterns ^1^.

	Tertiles of Nutrient Energy-Adjusted Intake			*p* for Trend
	T1 (Referent)	T2	T3 (Highest)	
Factor 1 (amino acid-carbohydrate nutrient pattern)				
*n* (ADHD/control)	103/93	104/94	89/109	
Model 1	1.00	0.99 (0.67, 1.48)	0.74 (0.50, 1.10)	0.220
Model 2	1.00	1.20 (0.74, 1.97)	0.89 (0.54, 1.46)	0.449
Factor 2 (vitamin-dietary fiber nutrient pattern)				
*n* (ADHD/control)	105/92	94/104	97/100	
Model 1	1.00	0.79 (0.53, 1.18)	0.85 (0.57, 1.26)	0.495
Model 2	1.00	0.87 (0.53, 1.41)	0.95 (0.58, 1.55)	0.842
Factor 3 (mineral-protein nutrient pattern)				
*n* (ADHD/control)	113/85	107/90	76/121	
Model 1	1.00	0.89 (0.60, 1.33)	0.47 (0.32, 0.71)	<0.001
Model 2	1.00	0.79 (0.49, 1.28)	0.53 (0.32, 0.88)	0.014
Factor 4 (fatty acid-cholesterol nutrient pattern)				
*n* (ADHD/control)	93/104	101/96	102/96	
Model 1	1.00	1.18(0.79, 1.75)	1.19 (0.80, 1.76)	0.631
Model 3	1.00	1.50(0.92, 2.46)	1.50 (0.91, 2.47)	0.181

Models 1 and 2: from conditional logistic model. Model 1: without further adjustment; Model 2: covariates adjusted for other potential confounding factors, including BMI, energy intake, family history of ADHD, household composition, maternal smoking during pregnancy, maternal drinking during pregnancy, paternal smoking before childbirth, paternal smoking after childbirth, maternal abortion history, maternal age at childbirth, parents’ education, household income, labor or delivery complications, birth weight, twin, full-term pregnancy, breast feeding, emotional abuse, and family conflicts; ^1^: ORs (95% CI) for all such values.

**Table 6:** Odds ratio (95% CIs) of ADHD for blood elements.

Blood Elements	Model 1				Model 2			
	OR ^1^ (95% CI)	*p*	OR per SD (95% CI)	*p*	OR (95% CI)	*p*	OR per SD (95% CI)	*p*
Zinc (μmol/L)	0.96 (0.96–0.99)	0.005	0.74 (0.60–0.90)	0.002	0.98 (0.96–0.99)	0.008	0.73 (0.60–0.89)	0.003
Iron (mmol/L)	1.16 (0.90–1.49)	0.243	1.22 (0.99–1.49)	0.073	1.11 (0.83–1.47)	0.492	1.19 (0.98–1.46)	0.086
Calcium (mmol/L)	0.77 (0.49–1.21)	0.258	0.96 (0.80–1.14)	0.608	0.82 (0.18–3.68)	0.795	0.94 (0.78–1.12)	0.464
Copper (μmol/L)	1.02 (0.96–1.08)	0.531	1.09 (0.89–1.33)	0.399	1.03 (0.96–1.10)	0.407	1.09 (0.89–1.33)	0.424
Magnesium (mmol/L)	0.78 (0.47–1.28)	0.320	0.98 (0.83–1.15)	0.763	0.74 (0.42–1.29)	0.286	0.98 (0.83–1.15)	0.804

Models 1 and 2: from conditional logistic model. Model 1: unadjusted; Model 2: covariates adjusted for other potential confounding factors, including BMI, energy intake, family history of ADHD, household composition, maternal smoking during pregnancy, maternal drinking during pregnancy, paternal smoking before childbirth, paternal smoking after childbirth, maternal abortion history, maternal age at childbirth, parents’ education, household income, labor or delivery complications, birth weight, twin, full-term pregnancy, breast feeding, emotional abuse, and family conflicts; ^1^: OR indicates likelihood of an ADHD.

## Abbreviations

The following abbreviations are used in this manuscript:

ADHD	attention deficit hyperactivity disorder
BMI	body mass index
CIs	confidence intervals
FFQ	food frequency questionnaire
ORs	odds ratios
PUFAs	polyunsaturated fatty acids
SD	standard deviation
24HR	24 h diet recalls

## Appendix

**Table A1:** Food groupings used in the dietary pattern analyses.

Food or Food Groups	Food Items
Grains	rice, millet, yellow rice, corn
Processed grains	bread, noodle, rice noodle, steamed roll
Red meat	pork, pig trotters, beef, beef brisket, mutton, and other meat
White meat	chicken, chicken paw, duck, duck feet, goose, and other poultry
Organ meat	pork, beef and mutton liver; chicken and duck giblets
Dairy products	cheese, margarine, yoghurt, skim and powder milk
Deep water fish	ribbonfish, yellow croaker, cuttlefish, snailfish
Freshwater fish	crucian carp, yellow catfish, silver carp, grass carp, rice field eel
Shellfish	shrimp, crab, shellfish
Fruits	banana, papaya, water melon, apple, honeydew, mango, pineapple, jackfruit, guava, orange
Tuber crops	sweet potato, dry sweet potato, cassava
Soya beans	soya bean, soya-bean milk, soya-bean flour, tofu, bean curd
Other beans	mung bean, red bean, broad bean, and other kidney beans
Root vegetables	carrot, white radish, turnip
Fruiting vegetables	pepper, eggplant, cucumbers, tomato, white gourd, pumpkin, cucumber, sponge gourd, bitter melon, courgettes
Leafy vegetables	cabbage, broccoli, greens, cauliflower, celery, water spinach, lettuces, spinach, chive, leek
Tuber vegetables	asparagus lettuce, bamboo shoots, water chestnut, lotus root, yams, taro, ginger, garlic, onion
Fungi and algae	mushroom family, seaweed
Snacks	potato chips, bread, cake, biscuits, other starchy snacks, pork floss
Fast food	convenience foods, frozen foods
Ice cream	ice cream
Drinks	cola, sprite, juices and juice drinks, vegetable juice drinks, milk drinks, chocolate soy milk, almond milk, tea beverage
Animal fats	lard
Vegetable oils	mixed oil, peanut oil, tea oil, olive oil, soybean oil, other edible oil
Condiments	soy sauce, vinegar, broad bean butter, pepper sauce, preserved bean curd, pickles, pepper, wild pepper, chili powder, salt, monosodium glutamate, others
Eggs	egg, duck’s egg, preserved egg, other eggs
Nuts	walnut, cedar nut, hazelnut, peanut, seeds of sunflower, watermelon and pumpkin
Sweets	candy, chocolate, preserved fruits, candied Chinese date
